# Central America Field Epidemiology Training Program (CA FETP): a pathway to sustainable public health capacity development

**DOI:** 10.1186/1478-4491-6-27

**Published:** 2008-12-16

**Authors:** Augusto López, Victor M Cáceres

**Affiliations:** 1 Division of Global Public Health Capacity Development, Coordinating Office for Global Health, Centers for Disease Control and Prevention, 1600 Clifton Road MS E-93, Atlanta, GA 30333, USA

## Abstract

The Central America Field Epidemiology Training Program (CA FETP) is a public health capacity-building training programme aimed at developing high-caliber field epidemiologists at various levels of the public health system. It began in 2000 as part of the effort to rebuild public health infrastructure in six Central American and Caribbean countries following the devastation of Hurricanes Mitch and Georges in late 1998. Since then, the CA FETP has evolved from one regional training programme managed by CDC to several national FETPs with each country assuming ownership of its domestic programme. The curriculum is competency-based, and is divided into a three-tiered training pyramid that corresponds to the needs at the local, district and central levels of the health system. Trainees at each tier spend about 20% of their time in the classroom and 80% in the field implementing what they have learned while being mentored by graduates of the programme. FETP trainees have responded to multiple natural disasters and conducted hundreds of investigations including surveillance evaluations, outbreak responses and planned studies. Also graduates of the CA FETP are assuming influential positions in their respective ministries. As countries meet the challenge of institutionalizing their programmes, the CA FETP concept will increasingly be recognized as a model for sustainable public health capacity development.

## Review

In late 1998 two hurricanes, Mitch and Georges, struck widespread areas of Central America (CA) and the Caribbean region, killing thousands of persons and causing extensive damage to public infrastructure. The health impact of this natural disaster underscored the lack of preparedness of governments in the region to react to a major public health emergency. Responding to this public health crisis, the United States Centers for Disease Control and Prevention (CDC) in collaboration with the United States Agency for International Development (USAID), the American Association of Public Health Laboratories (APHL), the Pan American Health Organization (PAHO), and ministries of health (MOH) in the region implemented a series of programmes to increase the capacity of the MOHs to respond to disease priorities and develop effective health information systems. Part of this investment prioritized training and service through the development and implementation of a regional Field Epidemiology Training Program (FETP).

The goal of the regional FETP (from here on referred to as CA FETP) is to build public health epidemiological capacity through training personnel to become high-caliber field epidemiologists and strengthening disease surveillance. The CDC has been a key technical partner, providing scientific and programmatic support for the CA FETP. In this article we describe the characteristics and evolution of this training programme, which is unique in its history, structure and implementation.

The CA FETP initiative includes five countries in Central America (Costa Rica, El Salvador, Guatemala, Honduras, Nicaragua) and the Dominican Republic. The training programme was modeled after the highly successful CDC Epidemic Intelligence Service (EIS) programme, a two-year training programme that has strengthened disease surveillance and response in the United States for over 55 years [1]. The EIS has been a model for more than 30 FETPs around the world, including the first one, initiated in Thailand in 1980 [2]. Each country has adapted the educational approach to its own unique needs. The CA FETP was designed to address the health issues present in the region and function effectively in the various political systems. The CA FETP was initially built as a two-year, master's (MPH) degree-accredited, training-in-service programme in field epidemiology; this is the highest or advanced level of training. In addition, because of the urgent need for field epidemiologists at all levels of the public heath system, two additional training tiers were added (to form a three-tiered or "pyramid" programme) to build capacity at local, district and central levels of the health system.

In the initial years (2000–2002), the programme was financed by USAID, as part of the Post-Hurricane Georges and Mitch Reconstruction Project, and accredited regionally by the *Universidad Autonoma de Nicaragua *(UNAN) in Managua. Subsequently, during 2003–2005, the programme was financially supported through in-country USAID funds and known as the *Servicio de Investigacion Epidemiologia y Vigilancia de Centro America, la Republica Dominicana, y Haiti (SIEVCADH) Project*. SIEVCADH was a follow-on to the Post-Hurricane Georges and Mitch Reconstruction Project and had the goal of continuing to strengthen epidemiological practice, effective data use and public health surveillance through a self-sustaining regional FETP. Though initially included in the project, efforts in Haiti ceased because of the adverse security situation at that time. During the third phase (2006 to the present), also known as the transition period, the CA FETP is evolving into several national FETPs financed primarily through country-acquired funds with the individual MOHs taking full responsibility for programme institutionalization. This transition was begun in response to an evaluation of the regional CA FETP in 2005 that concluded that the programme, though initially effective for rapidly increasing the number of field trained epidemiologists, was not sustainable in its then-current form.

## Training model

**Figure 1:**
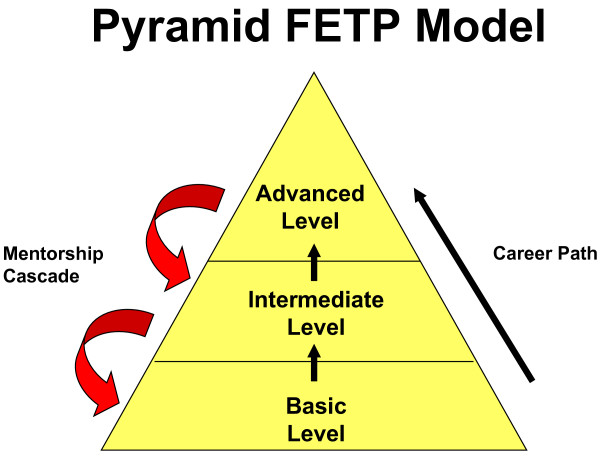
Conceptual model of the pyramid training approach used in Central America.

The FETP approach is training through service, i.e. learning by doing. We will use the term "FETP" to refer not only to the advanced level, but also to the entire three-tiered programme unique to Central America. The three-tiered concept (Figure 1), which has varied somewhat in its implementation in each country, is now evolving into a standardized and integrated approach, with a vision that all countries share a common set of core competences at each tier.

In the first tier, local health workers are trained in basic epidemiological methods enabling them to better respond to local health events and priorities. The first-tier training is conducted over a period of three to five months.

The second, more complex, intermediate tier is conducted over a period of nine months with participants being awarded a certificate by a university. Trainees for the first and second tiers generally gather in the classroom for three-day modules once each month and conduct their fieldwork during the intervening periods.

**Figure 2:**
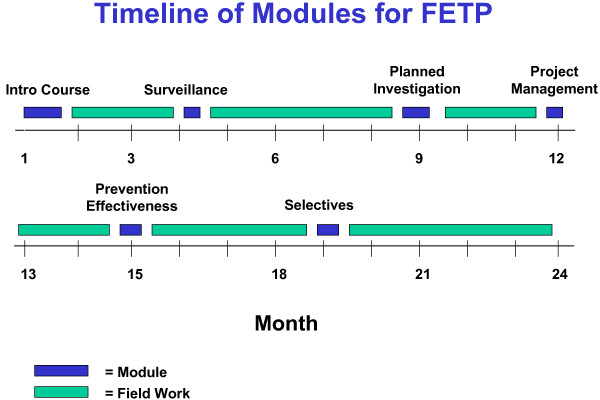
General time line for modules conducted by the Central American Regional FETP (advanced tier).

The advanced, two-year FETP (third tier) includes a three-week introductory module and several one- to two-week modules, totaling nine weeks or about 360 hours (Figure 2), with an oral defense of a major research project required to receive the master's degree (MPH).

The training at all three tiers is conducted in both the classroom and, most importantly, in the field, where trainees develop their skills and competences while performing the day-to-day duties of a field epidemiologist (surveillance, data analysis, outbreak investigation, etc.). The proportion of time devoted to each activity emphasizes the field (80%) over the classroom (20%). Participants in the FETP continue to get MOH salary support, because their fieldwork is considered to be part of their day-to-day responsibilities within the MOH.

Because of the in-service nature of the training, tutors (i.e. mentors) play a vital role. The design of the FETP pyramid enables a cascade of mentorship in which trainees in higher tiers serve as mentors to those in lower ones. The recommended tutor-to-trainee ratio is one-to-one at the advanced tier, one-to-two at the intermediate tier, and one-to-five at the basic tier. Mentors for all tiers generally receive training in mentorship skills prior to serving in their role, though countries vary in the extent to which this is implemented. Mentorship by CDC consultants was especially important in the early years when the first cohorts were being trained.

The curriculum of the three-tiered training programme is based on fundamental competences of a field epidemiologist [3]. It was constructed incorporating feedback and experience in the region, using the methodological expertise of an instructional designer. The competence areas are: epidemiological methods, biostatistics, public health surveillance, laboratory and biosecurity, communication, management and leadership, computer technology, learning and mentorship for public health professionals, epidemiology of priority diseases and injury, and emergency preparedness and response. Instructional materials for several of these competence areas have been developed in collaboration with the North Carolina Institute for Public Health at the University of North Carolina School of Public Health at Chapel Hill.

The curriculum (core and elective topics) is delivered primarily in the classroom, and there are homework assignments between modules. There is an increasing interest in the use of innovative self-instructional and distance-based learning (CD-ROM or Web-based) methods to complement the classroom training and provide more learning options. However, since Web-based learning is a fairly new approach in the region, incorporating this modality into the FETP will be gradual and carefully evaluated.

FETPs around the world vary as to whether or not they incorporate an academic degree, typically a master's (MPH) degree. When the FETP was being planned in Central America, the countries expressed their desire for master's degree accreditation due to the importance of a degree for professional advancement. During the transition period, accreditation for the CA FETP has been moved from UNAN in Nicaragua to the University of del Valle in Guatemala (UVG), which also accredits the Guatemalan national FETP. Since 2006 countries have been increasingly taking ownership of their respective national FETPs and negotiating with local universities. Costa Rica was the first country to achieve accreditation of its national FETP by a local university, and similar initiatives are ongoing in El Salvador and Honduras. Because of this transition, as of 2007 we no longer speak simply of one regional programme. Rather, there is one national programme (Guatemalan FETP) that reaches out regionally by accepting trainees from other countries (some with less mature programmes). In addition there are several countries that together with Guatemala have formed a regional coalition of independent, mutually supporting national FETPs. In addition to planning joint initiatives, they support each other through sharing materials and experiences.

## Outcomes and impact

CA FETP trainees and graduates have improved health policies and contributed to the strengthening of health systems in their countries. During the period 2001–2007, the CA FETP graduated four advanced-tier cohorts (58 trainees). Achievements by trainees during these years include 181 surveillance systems evaluated, 222 outbreaks identified and investigated, and 167 research studies implemented. In Guatemala and El Salvador, where the training pyramid has been in effect the longest, there have been 755 graduates from the basic tier (630 and 125, respectively) and 255 graduates from the intermediate tier (125 and 130, respectively). In Guatemala, El Salvador and Honduras the FETP has played an important role in the reorganization of national epidemiology offices. A FETP trainee project in El Salvador led to the implementation of a new National Injury Surveillance system. FETP investigations on chronic renal failure in sugar cane workers in Nicaragua led to changes in national labour policies.

CA FETPs have also been active in responding to national emergencies. During the 2001 earthquake in El Salvador, all the trainees of the first regional cohort supported the El Salvador MOH in various activities related to the disaster (e.g. needs surveys, implementing surveillance systems, investigating outbreaks). FETP trainees also responded to recent disasters in Guatemala (Hurricane Stan, 2006) and Honduras (Hurricane Felix, 2007). During Hurricane Stan there was a dramatic correlation between the quantity and quality of surveillance data reported from an affected health area and the presence of an intermediate-tier FETP graduate. In addition to natural disasters, there have been national-scale outbreaks in which FETP trainees played a key role, such as those due to dengue (Costa Rica, Dominican Republic, El Salvador, Guatemala); cholera (El Salvador); vaccine-derived poliovirus (Dominican Republic); hepatitis C (Nicaragua), pertussis (Costa Rica); and pesticide poisoning (Dominican Republic, Guatemala, Nicaragua).

A measure of success for the programme is the number of graduates that remain working within the health system in their country (Table 1). Of current advanced-tier FETP graduates, 80% still serve within their country's MOH, 5% serve in international positions and 15% serve in other institutions or are retired. Graduates of the CA FETP lead national epidemiology offices in the MOHs of Guatemala and Honduras. Some of the graduates have been contracted as regional consultants for CDC programmes (Global AIDS Program, Avian Influenza Program). A CA FETP graduate from El Salvador is the current chairman of TEPHINET (Training Programs in Epidemiology and Public Health Interventions NETwork), the global network of FETPs.

**Table 1:** Present positions of advanced-tier FETP graduates in Central America region, n = 58.*

**Country**	**Ministry of Health**			Other Institutions**	**International health-related organizations**	**Retired**	**Total**
	**Epidemiology office**	**Other MOH programmes**	**Local and district**				
	
Costa Rica	6		1				7
El Salvador	6				2	1	9
Guatemala	6		3	1	1	1	12
Honduras	5	1	2		1		9
Nicaragua	3		3	1	1	1	9
Dominican Rep.	5	3	2	1			11
Panama***	1						1
Total	32	4	11	3	5	3	58
**Percentage**	**55%**	**7%**	**19%**	**5%**	**9%**	**5%**	**100%**

*Data current as of 2007.

**Other institutions include other governmental or nongovernmental institutions, such as nongovernmental organizations (NGOs) and universities.

***Panama is included because there was a trainee residing there who received technical and financial support from the CDC Global AIDS Program.

One strategy of the CA FETP is to promote the career path of the epidemiologist by demonstrating the impact of a network of field epidemiologists collaborating as teams throughout various levels of the ministry of health. The National Center for Epidemiology in the Guatemala MOH has designed a project to implement field epidemiologist networks in its health zones, based on the three tiers of the FETP pyramid. The FETP trainees (or graduates) of the first, second and third tiers will work at the local, district and zonal levels, respectively, and meet periodically as a team to analyse surveillance and conduct outbreak investigations and other public health interventions. Standardized evaluations are being planned before (baseline) and after implementation of these networks in each province to demonstrate their impact on public health practice.

Two other networks deserve mention. One of them is represented by the Regional Technical Committee (RTC), composed of national epidemiology directors and FETP coordinators. The leadership and political support shown by the RTC for the programme have increased substantially over the past two years. The RTC is led by a rotating chairperson (appointed annually) and oversees the development of the programme regionally. Monthly meetings are held using Internet-based conferencing technology and there is an annual meeting coordinated by the Pan American Health Organization, at which a workplan is developed and agreements are signed. The other network, mentioned above, is TEPHINET [3] which has hosted annual conferences and given opportunities for CA FETP trainees to give their presentations in an international forum. Most of the more than 200 international oral and poster presentations given by CA FETP trainees and graduates have been at the three global and four regional TEPHINET scientific meetings that have taken place since 2001.

## Conclusion

There has been great progress in the evolution of the regional CA FETP into a coalition of national FETPs, but important challenges remain. Though the countries share a common history, culture and language, the political cycles in each country vary, and each political change is often accompanied by turnover in health authorities that oversee the national FETPs. In establishing each national FETP, each country must negotiate its own domestic accreditation agreement while conforming to a regional standard with regard to competences and field supervision.

One of the key challenges of the CA FETP is the long-term sustainability and institutionalization of FETPs in each country. For this to occur, each national programme must ideally be financed by its inclusion as a line item in the MOH budget. External donors may continue to play an important role in providing support to FETPs, but in this period of transition, it is now individual countries that conduct these negotiations and agreements. Along with national financing, other indicators on the path to sustainability are: (1) the inclusion of the FETP in the organigram of the MOH; (2) the presence of a full-time FETP director; (3) well-defined job functions that allow epidemiologists within the MOH to practice skills acquired during training; and (4) a career ladder that rewards advanced training in field epidemiology.

The CA FETP has recently been promoting the concept of academic networks. As each country moves forward to establish accredited programmes with local universities, it will be important that these universities collaborate to facilitate adherence to a core curriculum and exchange of professors/tutors, trainees and course credits. In this manner, lessons will be shared and synergies created. We see great potential for the Internet as a medium for communication and delivery of course content. Over the next year the CA FETP will be pilot-testing and evaluating training modules administered over the Internet. If this roll-out is successful, it will greatly enhance each country's range of options for training in field epidemiology and enhance the sustainability and caliber of national programmes.

TEPHINET and the RTC will continue to play a vital networking role. In addition to enhancing the scientific rigour of the programme, the annual TEPHINET meetings will continue to serve as an important forum for the countries in the CA FETP to exchange experiences with other FETPs around the world. We have recently seen increased interest by FETPs in South America and Asia in establishing their own "training pyramids." At a recent leadership workshop in Guatemala City, members of the RTC met with representatives from the four FETPs in South America (Argentina, Brazil, Colombia and Peru) and discussed a broader collaboration to enhance regional sustainability of all FETPs.

In conclusion, the CA FETP has made an important contribution on field epidemiology practice in the Central American region. As countries continue to take full ownership of their national programmes and institutionalize them, the CA FETP experience will increasingly serve as a model for sustainable public health capacity development in the region and beyond.

## Competing interests

The authors declare that they have no competing interests.

## Authors' contributions

Both authors contributed to the conceptualization, drafting and finalization of this manuscript.
